# Evolving priorities in health and social care for people with learning disabilities: Tracking the emergence of dementia as a priority and policy focus in the UK (2015–2023)

**DOI:** 10.1002/alz70860_099730

**Published:** 2025-12-23

**Authors:** Tamlyn J Watermeyer, John‐Joseph Russell, Jaheeda Gangannagaripalli, Sue Molesworth, Smruti Bulsari

**Affiliations:** ^1^ Edinburgh Dementia Prevention, Centre for Clinical Brain Sciences, College of Medicine and Veterinary Medicine, University of Edinburgh, Edinburgh, United Kingdom; ^2^ University of Northumbria, Newcastle upon tyne, England, United Kingdom; ^3^ National Institute of Health Applied Research Collaboration North East & Cumbria, Newcastle‐upon‐Tyne, England, United Kingdom; ^4^ Faculty of Health & Life Sciences, Northumbria University, Newcastle‐Upon‐Tyne, England, United Kingdom; ^5^ The University of Manchester, Manchester, England, United Kingdom; ^6^ National Institute of Health Applied Research Collaboration Greater Manchester, Manchester, England, United Kingdom; ^7^ Keele University, Keele, England, United Kingdom; ^8^ National Institute of Health Applied Research Collaboration East of England, Essex, England, United Kingdom; ^9^ University of Essex, Colchester, Essex, United Kingdom

## Abstract

**Background:**

Individuals with learning disabilities face significant disparities in health and social care, through systemic barriers limiting access to services and impacting their quality of life. UK policies and reports aim to address these inequities, but little is understood about how health and care priorities for this population have evolved over time. Notably, dementia's emergence as a growing concern for people with learning disabilities remains underexplored, despite its increasing prevalence within this group.

**Method:**

This study utilised qualitative content analysis, supported by Natural Language Processing (NLP), to systematically review key health and social care policy documents from 2015 to 2023. Analysed documents include *Learning from Lives and Deaths – People with a Learning Disability and Autistic People* (LEDER, 2016–2022), *Building the Right Support* (2015, 2022), *Right Support, Right Care, Right Culture* (2015, 2022), *Learning Disabilities: Health Policies* (2023), and relevant *National Institute of Care Excellent (NICE)* guidance on mental health in this population. Text from these reports was cleaned and pre‐processed before word frequencies were identified. Word frequencies were bigrams and trigrams, defined as occurrence frequencies of two‐word and three‐word combinations, respectively.

**Result:**

Analysis of bigrams and trigrams revealed evolving priorities for people with learning disabilities across documents. Frequently occurring terms, such as “mental health”, “social care” “building right support,” signalled a consistent focus on addressing health inequalities and improving access to social care. A temporal dimension was observed, with earlier reports primarily addressing systemic failings and barriers to access, whereas more recent documents emphasised person‐centred care, workforce competence, and individualised support. Despite this progress, dementia‐related terminology did not emerge as a growing focus in most reports, with only NICE and LEDER explicitly addressing dementia.

**Conclusion:**

Although data synthesis is ongoing, the study highlights the dynamic evolution of health and social care priorities for people with learning disabilities. It identifies significant progress in addressing inequities while exposing persistent gaps, particularly in integrating dementia care into existing strategies. The findings underline the need for sustained focus on dementia within policy frameworks and demonstrate NLP's value in tracking policy trends and informing targeted interventions to ensure equitable, person‐centred care across the lifespan.

